# Environmental Enrichment Reduces Signs of Boredom in Caged Mink

**DOI:** 10.1371/journal.pone.0049180

**Published:** 2012-11-14

**Authors:** Rebecca K. Meagher, Georgia J. Mason

**Affiliations:** Animal and Poultry Science Department, University of Guelph, Guelph, Ontario, Canada; Federal University of Parana (UFPR)) – Campus Palotina, Brazil

## Abstract

Animals housed in impoverished cages are often labelled ‘bored’. They have also been called ‘apathetic’ or ‘depressed’, particularly when profoundly inactive. However, these terms are rarely operationally defined and validated. As a negative state caused by under-stimulation, boredom should increase interest in stimuli of all kinds. Apathy (lack of interest), by contrast, should manifest as decreased interest in all stimuli, while anhedonia (loss of pleasure, a depressive symptom) should specifically decrease interest in normally rewarding stimuli. We tested the hypotheses that mink, a model carnivore, experience more boredom, depression-like apathy, or anhedonia in non-enriched (NE) cages than in complex, enriched (E) cages. We exposed 29 subjects (13 E, 16 NE) to ten stimuli categorized *a priori* as aversive (e.g. air puffs), rewarding (e.g. evoking chasing) or ambiguous/neutral (e.g. candles). Interest in stimuli was assessed via latencies to contact, contact durations, and durations oriented to stimuli. NE mink contacted all stimuli faster (P = 0.003) than E mink, and spent longer oriented to/in contact with them, albeit only significantly so for ambiguous ones (treatment*type P<0.013). With stimulus category removed from statistical models, interest in all stimuli was consistently higher among NE mink (P<0.0001 for all measures). NE mink also consumed more food rewards (P = 0.037). Finally, we investigated whether lying down while awake and stereotypic behaviour (both increased by NE housing) predicted these responses. Lying awake positively co-varied with certain measures of increased exploration. In contrast, stereotypic ‘scrabbling’ or locomotion (e.g. pacing) did not. Overall, NE mink showed no evidence of apathy or depression, but instead a heightened investigation of diverse stimuli consistent with boredom. This state was potentially indicated by spending much time lying still but awake (although this result requires replication). Boredom can thus be operationalized and assessed empirically in non-human animals. It can also be reduced by environmental enrichment.

## Introduction

Boredom, apathy and depression are often hypothesized to occur in animals housed in impoverished environments (e.g. [1]–[3]). However, in very few cases has the use of these terms been validated empirically, and often no precise definitions are given. The three terms have also been used in overlapping ways to describe non-human animals, which can lead to confusion. For example, Wood-Gush and Beilharz [4] suggested piglets in non-enriched environments were bored, but Wood-Gush and Vestergaard [5] described the very same piglets as apathetic, largely based on the same behaviour: the subjects were more inactive and showed less behavioural diversity than piglets given enrichment. However, these states have distinct definitions and different clusters of symptoms in humans [6], which allow them to be distinguished from one another behaviourally.

Depression is the only one of these three states that has been clearly operationalized and demonstrated, at least in part, in non-humans. Because chronic stress, and more specifically, exposure to uncontrollable stressful events (reviewed by Henn and Vollmayr [7]), is believed to be one of its major causes, the chronic stress and lack of control known to exist in many captive environments may well induce similar states in non-human animals (see [2], [8]). Depression is a complex phenomenon with several subtypes (see [9]); reviewing and assessing all aspects of the disorder is beyond the scope of this paper. However, one of its core symptoms, anhedonia, is of interest here; it is common to all subtypes and regularly assessed in animal models (cf. [10]). Anhedonia is defined as a reduced capacity to experience pleasure, typically measured in terms of the decreased consumption of rewards (e.g. sucrose for rodents: [11]). Apathy is similarly a common symptom of depression, although it can also stand alone as a disorder (reviewed in [12]). Like depression, it is sometimes a response to uncontrollable stress [13]. It is thought of as a lack of interest or concern, but in practice, it is typically operationally defined as a state of generally reduced motivation or participation in activities [14]–[16]. Thus, apathy should be expressed as a decreased motivation to obtain or interact with any stimuli, while anhedonia would decrease motivation for rewarding stimuli specifically. Depressed people whose symptoms include both anhedonia and a more general lack of emotional expression (indicative of apathy) typically exhibit decreased interest in obtaining stimulation [17].

Boredom has never been empirically demonstrated in animals, because it is difficult to define operationally (see [18]). In humans, it can be defined as a negative affective state caused by under-stimulation or monotony [19]. (Recent work aims to establish a more inclusive definition, since the same affective state can have internal causes in humans, such as an inability to focus attention [20]; however, our focus here is on this extrinsically-caused form of boredom.) It has been studied in a range of situations, from complete sensory deprivation to the performance of very monotonous tasks (reviewed by [19]). The situation of prisoners serving life sentences probably best parallels the one faced by captive animals; neither prisoners nor animals are deprived of all stimuli, but they do face a very unchanging, inescapable environment, which induces boredom in the humans (see [21]). In all of the above cases, people report that the situation is aversive [22]. Thus, bored people are motivated to seek novel stimulation (e.g. bungee jumping: [23]; novel foods: [24]; recreational drugs: [25]), performing activities collectively known as ‘sensation seeking’ [26]. Impoverished housing may similarly increase motivation to obtain general stimulation in rodents; for example, it can increase instrumental responses to obtain amphetamine in rats (e.g. [27]). Since we cannot rely on verbal self-reports for non-humans, this motivation to obtain general stimulation must form the basis of any operational measures of boredom (as in [28]). Indeed, the concept of boredom has often been used as an explanation for why animals explore, and thus been considered synonymous with motivation to obtain novel stimulation (see [29], [30]; but cf. Wemelsfelder [31], who conceptualized it differently).

In addition to predicting different levels of interest in stimuli, these three states also differ in their predicted behavioural correlates. In humans, symptoms of boredom vary, ranging from lethargic inactivity to active responses such as restlessness and stereotypic behaviour (SB) (reviewed by [19], [22]). Similarly, in non-humans, boredom has been posited to be linked to some forms of inactivity (e.g. [3], [4], [32], [33]), but also to play a role in the development of SB [18], [31], [34]. By contrast, only inactive or unresponsive animals are typically labelled ‘apathetic’ or ‘depressed’ (e.g. [1], [2], [35]). While depression does not always lead to increased inactivity in humans, certain forms of it do (see [36]), and the animal models based on response to chronic stress tend to be associated with inactivity (e.g. [37], [38]).

We investigated how these states could be differentiated from one another in non-humans using captive mink (*Neovison vison* [Schreber, 1777]) as a model. Their welfare was manipulated by housing them in either non-enriched (NE) or enriched (E) cages. NE cages were similar to, although larger than, typical fur farm cages in most of the world; the provision of simple enrichments is currently required only in Northern European countries. The E cages were larger and provided more stimulation and/or behavioural opportunities, since they included wading water and a variety of manipulable objects, both types of enrichment validated for use with mink [39], [40]. These specific enriched cages had previously been shown to be valued resources [41], to improve welfare, and to have clear effects on home cage behaviour [42]–[44].

This experiment had two aims. The first was to test the alternative hypotheses that mink in NE cages are bored, or that they are apathetic and/or anhedonic. To do so, we compared their responses to a wide range of stimuli to those of mink housed in enriched (E) cages, who should experience lower levels of these negative affective states. We predicted that if bored, non-enriched mink would show evidence of increased interest in all stimuli presented; if apathetic, they would show decreased interest in all stimuli; and if anhedonic but not apathetic, they would show decreased interest in rewards only (see Table 1). The second aim was to determine whether spontaneous home-cage behaviour could be used as an indicator of the relevant psychological state. Thus, we tested whether any observed differences in expressed interest in stimuli correlated positively with SB, or with lying awake but inactive [43], [44]: two behaviour patterns previously hypothesized to reflect boredom, apathy or depression; elevated by NE housing in this species; and also known not to reflect fear or anxiety in mink (states not of interest here). If NE mink were bored, it was predicted that both types of behaviour would correlate positively with our ‘interest’ indicators, while only lying awake was expected to correlate with interest in stimuli (and negatively so) if NE mink were depressed or apathetic.

**Table 1:** Interest in different types of stimuli depending on psychological state.

State	Aversive	Neutral	Rewarding
Apathy	Low	Low	Low
Anhedonia	Normal	Low	Low
Boredom	High	High	High

## Methods

### Ethics statement

This research was conducted in accordance with the guidelines of the Canadian Council on Animal Care. It was approved by the institutional animal care committees of the University of Guelph (AUP 07R033) and Michigan State University (AUF 09/09-136-00).

### Animals and housing conditions

The subjects were 29 captive-bred Black mink on the Michigan State University research farm. They were housed indoors on a natural light cycle. All subjects were approximately ten months old, and therefore had just reached sexual maturity. For approximately seven months, they had been housed differentially in either enriched (N = 6 male, 7 female) or non-enriched conditions (N = 8 male, 8 female). Non-enriched (NE) mink had 75 (L) ×60 (W) ×45 (H) cm wire-mesh cages, with a nest box on the front. Enriched (E) mink had identical home cages, but also had access, via a wire-mesh overhead ‘tunnel’, to a cage of double that width (details in [43]), that contained running water in a small trough to allow wading and head-dipping, shelf-like structures, and manipulable objects (e.g. rubber dog toys). New objects were added to this enriched cage every month. After long-term exposure, these differential housing conditions have been confirmed to result in lower levels of physiological stress in E mink compared to NE mink [42].

### Spontaneous behaviour

Immediately before testing, baseline home-cage behaviour was observed live for seven days, using a modified form of instantaneous scans in which the observation lasted up to 15 seconds if necessary to identify the behaviour (see also [43]). Scans began at 08:00 and continued throughout the pre-feeding (active) period every 15 minutes until 12:00. The behaviour patterns of interest recorded were lying still but awake (eyes visibly open) and two forms of SB: ‘scrabbling’ (repetitive scratching at a wall of the cage or nest box, a form that is not commonly reported in other carnivores [43]) and locomotor SB, defined as three or more repetitions of a movement involving the whole body or upper body.

### Interest in stimuli: investigatory behaviour tests

Two types of test were used to assess interest in stimuli: tests in which stimuli of various types were placed on or near the cage, and consumption tests in which rewards were offered in the cage. The first type consisted of a series of ten tests, conducted over eight days in late February. In each test, an auditory cue was given to signal the start of a test; this encouraged E mink to return to the home cage if they were elsewhere. A stimulus was then placed on top of or in front of the cage. The stimuli were categorized *a priori* as aversive, rewarding, or ambiguous/neutral (neutral in the sense of lacking clear biological relevance). The specific stimuli are listed in Table 2, along with rationales for their categorization and results used to test the validity of this categorization *post hoc*. Observations began as soon as the mink oriented to the stimulus, and continued for five minutes. The indicators of ‘interest’ were the total duration of orientation to the stimulus, duration of contact with the stimulus (a subset of time oriented), and latency to make contact. Orientation was defined as the head being pointed towards the stimulus with the eyes open, and contact as the nose or front paws of the mink touching the stimulus (contact with any other body part was never prolonged and appeared accidental). Most studies of interest in or reactivity to stimuli use one or two of these measures: duration or frequency of contact is the standard measure of exploration [45] and latency to approach is a validated measure of motivation (e.g. [46]), while orientation is a common and appropriate measure of attention [47] that can provide greater insight into underlying states when used in conjunction with the others, as discussed below. Decreased latencies, along with increased duration of both orientation and contact, would indicate heightened interest, while the reverse would indicate decreased interest. There was a single stimulus, the predator silhouette, with which the mink could not make contact since it moved some distance above the cage; thus, only duration of orientation was assessed for that stimulus. Latencies were recorded live using a stopwatch, while video was used to obtain durations. If the mink never made contact with the stimulus, the maximum latency of 300 s was assigned.

**Table 2:** Order in which stimuli were presented in investigatory behaviour tests, with validation of the categories to which they were assigned.

Stimulus	Type	Order of presentation	Rationale	Sum of fear scores
Air puff	Aversive	5	Aversive to many species (e.g. Huot et al., 2001: rats; Lansade and Simon, 2010: horses)	20
Predator silhouette (eagle)	Aversive	7	Natural predator (Dunstone, 1993); may be innately frightening (cf. Brown et al., 1992)	7
Handling glove	Aversive	2	Associated with past handling experiences, typically stressful; shown to elicit fear in some individuals (Meagher et al., 2011)	22
Predator odour (bobcat urine)	Aversive	10	Natural predator (Dunstone, 1993); may be innately frightening (cf. Blanchard et al., 1990)	2
Plastic bottle	Ambiguous	1	Novel and no apparent biological relevance	3
Maraca	Ambiguous	6	Novel and no apparent biological relevance	0
Peppermint scent	Ambiguous	4	Novel and no apparent biological relevance	0
Ocean scented candle	Ambiguous	8	Novel and no apparent biological relevance	0
Moving toothbrush	Rewarding	3	Known to elicit prolonged chasing; can be used as a reward to elicit operant responses (unpublished data)	1
Female faeces*	Rewarding	9	May be attractive to males during mating season, when test was conducted	0

* Presented to males only.

Fear was considered a possible confounding factor, because environmental enrichment is known to decrease fearfulness in many species (e.g. [48], [49]), and fear might inhibit investigation of novel stimuli, thus making non-enriched animals appear less interested in them. We therefore controlled for differences in fear in two ways. First, we used multiple measures of interest in stimuli which would not be influenced by fear in the same direction: animals that were fearful would be expected to show increased latencies, but not decreased orientation, since fear and anxiety are associated with vigilance, but immobility or avoidance rather than approach ([50]; cf. [51], [52]). Second, we scored individuals on the presence or absence of four species-typical indicators of fear: retreat, alternation between retreat and withdrawal, screaming, and spraying a stress odour (reviewed by [51], [53]). This was summed to a score out of four for each test, and used to test for effects of the housing treatments on fearfulness, as well as to validate the “aversive” category (see Table 2).

Tests alternated between the three stimulus types (see order in Table 2). Stimuli from each category were equally divided between the morning (beginning at 08:30) and afternoon (beginning at 14:00) sessions. When aversive stimuli were presented in the morning, no other test took place that day, to allow the mink time to recover if stressed. All tests took place in the home cage; if E mink would not return to the home cage to begin a test, it was not conducted because competing motivations to use the enrichments tended to distract the mink from attending to the test stimulus when in the enriched cage. If mink were asleep and did not awaken even when the experimenter tapped on the cage, the test was skipped and conducted later that day if possible. If mink oriented to the stimulus but then slept continuously for more than 180 seconds of the test, data for that test were excluded from analysis because they were judged not to accurately reflect the individual's typical level of interest (see [54] for evidence that in “drowsy” states, mink respond differently to stimuli).

### Interest in stimuli: food consumption tests

Interest in rewarding stimuli was also assessed as the number of food ‘treats’ (foods the mink are motivated to eat even when not hungry) that the mink consumed. Three treat types were presented in separate tests: wet cat food (Fancy Feast™ chicken hearts and liver), ferret treats (Bandits™ chicken flavour), and diced hot dog sausages. For each test, 30 pieces were given, and the number consumed in 15 minutes was recorded. Treats removed from the food dish and cached in the nest box were counted as consumed, as these were typically eaten shortly after the end of the test, while those dropped on the floor were not. All mink of each sex were given an equal amount of their regular food on these days, and the proportion left uneaten was scored visually to the nearest 5% to control for differences in appetite.

### Statistical analyses

Data were analysed using JMP 8 and 9 (SAS Institute Inc., NC, USA, 2009; 2010). A general linear model (GLM) was applied to each dependent variable (duration oriented, latency to make contact, duration of contact, and proportion of treats consumed), controlling for individual as a random factor, nested in sex and treatment, since the tests involved repeated measures. The effects of stimulus type (category), stimulus order, and all interactions were analysed. Stimulus order effects are not presented unless they interacted with the variables of interest. Where stimulus order had no linear effect that would indicate habituation or sensitization over the repeated tests, stimulus was instead included as a categorical variable. This was the case for the treat consumption tests. Initially, time of day (AM or PM) was included in the models, but because it was not a significant predictor of any variable of interest, it was removed. The models for treat consumption also included the proportion of the regular diet not eaten as a covariate. If, for any reason, the mink were not visible on the video for a portion of the test, duration of time oriented and duration in contact were calculated based on proportion of visible time. The analyses for the investigatory behaviour tests were also repeated with the stimulus category removed from the models.

The relationships of each measure of ‘interest’ with SB and time spent lying awake were assessed using another set of GLMs. Because repeated measures models could not be run using these continuous predictors, least square means for each measure of interest (e.g. latency) were first obtained from GLMs similar to those run above: one using data from all stimuli and a set of GLMs split by stimulus type. These means provided a single estimate of overall interest for each individual that adjusted for the effects of missing data, and removed the effects of sex and stimulus order. Treatment was not included in the model so that its effects could be investigated in the following analyses. These least square means were then used as the dependent variables in GLMs with treatment, proportion of scans on which the mink were performing the behaviour of interest, and their interaction, as the predictors. For treat consumption, a simple average was used in place of the least squares mean, since there were no missing data, and there had been no linear effect of stimulus order, making adjustments to the average unnecessary. These models therefore controlled for the effects of sex. Due to non-orthogonality of housing treatment and spontaneous home-cage behaviour in all of these models, sequential (Type I) sums of squares were used, with behaviour as the last main effect in the model.

Transformations were required to meet the assumption of normality in some cases: latencies were log-transformed, while the orientation data were squared for the repeated measures models. A logit transformation with a bias correction factor of 0.003 was applied to the proportion of treats consumed. A non-parametric Wilcoxon signed-rank test for matched pairs was used to determine whether fear scores were higher in tests with stimuli categorized as aversive than in any other tests, which would confirm that our choice of stimuli for each category was appropriate. Fear scores were also summed across all tests and these sums were compared between housing treatments using Wilcoxon rank-sums tests, since the data were non-normal, both split by sex and with the sexes pooled.

Due to multiple testing (which increases the chance of Type I errors), the Benjamini and Liu [55] procedure to control the ‘false discovery rate’ was applied for each main hypothesis (see also [56]). This procedure sets an α smaller than 0.05. However, effects with p-values greater than that determined by the false discovery rate procedure but less than or equal to the conventional level of 0.05 are still reported. Such effects are treated as pilot findings that need replication in the future.

## Results

### Validation of stimulus categories

Overt signs of fear occurred relatively infrequently. However, as expected, fear scores were higher for stimuli categorized *a priori* as aversive rather than ambiguous or rewarding (d.f. = 28, W = −162.5 and W = −175.5, respectively; both P<0.0001). In fact, while 26 of the 29 mink showed fear on at least one trial, only four of those had a fear score above zero on any ambiguous or rewarding trial. The predator odour was the only stimulus categorized as aversive that did not elicit more overt signs of fear than all stimuli from other categories (see Table 2 for fear scores by stimulus). There was no effect of housing treatment on fear scores, either pooled or split by sex (P>0.05). This evidence supports the categorization of these stimuli as aversive, with the possible exception of the predator odour.

**Figure 1:**
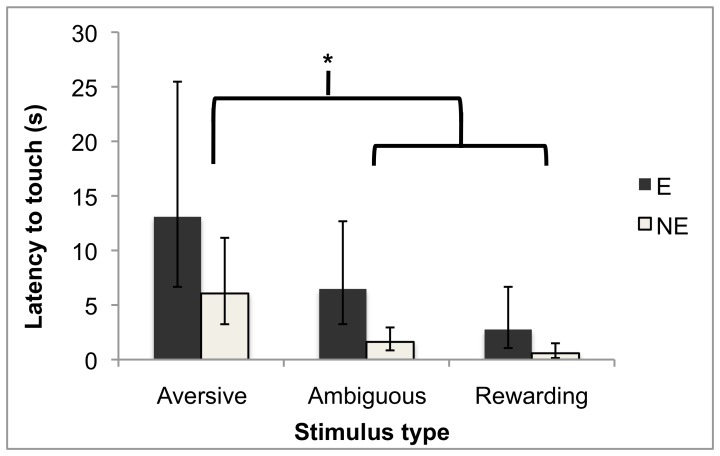
Latency to make contact with the stimulus, split by housing treatment and stimulus type. Data are back-transformed least squares means, with error bars indicating confidence intervals. * indicates a significant difference at the α = 0.05 level. There was also a significant main effect of housing treatment. A version of this figure based on earlier statistical models was included in Meagher [81].

Patterns of investigation also varied between our categories. Latencies to make contact differed significantly depending on stimulus category (F_2,207_ = 18.5, P<0.0001; Figure 1), being longest for aversive stimuli (Tukey's Honestly Significant Difference; see next section for interactions). Mink also spent more time in contact with stimuli *a priori* categorized rewarding than with the other two stimulus types (main effect of stimulus type: F_2,188_ = 44.0, P<0.0001; Tukey's HSD). Such effects were not very consistent at the level of individual stimuli, however. For example, looking at the two ‘rewarding’ stimuli for which these data were collected, it is apparent that the increased contact effect was driven only by the moving brush: E mink spent less time in contact with female faeces than they did with some aversive and ambiguous stimuli (Table 3). This may indicate that our categories were not perfect, or that investigatory behaviour is not closely related to stimulus valence. Data from other species reveal that animals can display much investigation of certain types of stimuli that they do not find pleasant (e.g. predator inspection [57]; males exploring stimuli from rival conspecifics [zebra finches working to see rivals [58]; Siamese fish working to display at a mirror [59]]). Thus the appropriate categorization of the female faeces stimulus is unclear (an issue returned to in the Discussion).

**Table 3:** Investigatory behaviour tests.

Stimulus	Type	Mean latency to touch ± SE (s)	Mean attention ± SE (s)	Mean contact ± SE (s)
Air puff	Aversive	10.4±2.6	171.8±7.1	116.3±7.1
Predator silhouette	Aversive	N/A	148.6±11.8	N/A
Glove	Aversive	89.2±18.9	151.6±11.9	65.5±10.8
Predator odour	Aversive	18.9±10.8	197.3±14.5	182.5±14.6
Bottle	Ambiguous	21.3±11.0	176.2±11.4	135.6±11.8
Maraca	Ambiguous	11.2±4.8	192.9±10.8	132.5±12.2
Peppermint scent	Ambiguous	37.8±15.3	153.4±16.4	93.0±12.2
Scented candle	Ambiguous	16.9±11.2	144.3±13.7	101.6±10.7
Moving toothbrush	Rewarding	1.5±0.3	273.6±2.4	223.4±5.6
Female faeces	Rewarding	12.5±6.8	148.7±16.7	136.3±16.9

The planned analyses blocking by stimulus category were still conducted. On average, responses to the different categories did differ, and so this allowed us to control for known sources of variation such increased latencies to approach initially aversive stimuli; furthermore, without such categories we could not test the anhedonia hypothesis. However, once it became clear that the anhedonia hypothesis was rejected (see next section), due to the possibility that at least two stimuli (female faeces; bobcat urine) were incorrectly categorized, we also re-investigated treatment effects on analyses of latency to contact, duration of contact and duration of orientation in models in which stimuli were not grouped into categories but treated as an independent nominal variables. The results of both of these types of analysis are described below.

### General interest in stimuli: housing treatment effects

#### Investigatory behaviour tests

**Figure 2:**
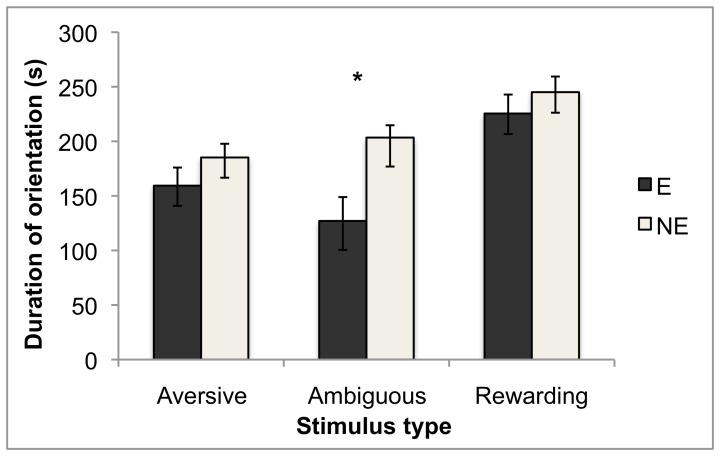
Duration oriented to the stimulus, split by housing treatment and stimulus type. Data are back-transformed least squares means, with error bars indicating confidence intervals. * indicates a significant difference at the group α = 0.05 level (Tukey's HSD). A version of this figure based on earlier statistical models was included in Meagher [81].

**Figure 3:**
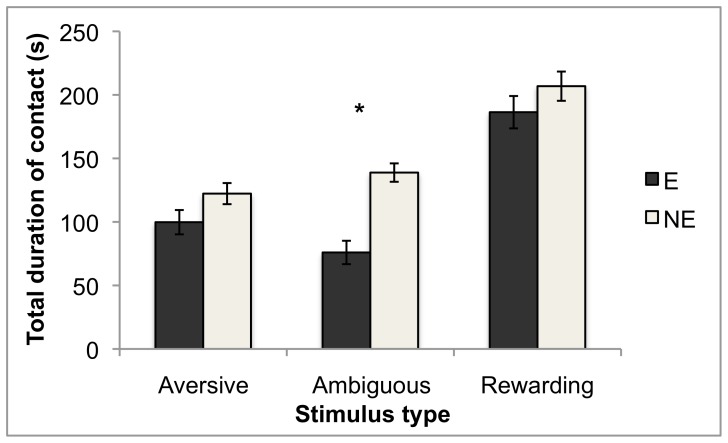
Duration in contact with the stimulus, split by housing treatment and stimulus type. Data are means ± standard error. * indicates a significant difference at the group α = 0.05 level (Tukey's HSD). A version of this figure based on earlier statistical models was included in Meagher [81].

Three mink had at least one test excluded because they slept for >180 s after the test began; all of these were E mink. For the remaining mink, when the stimulus categories determined *a priori* were included, housing treatment interacted with stimulus type for duration of orientation to stimuli (F_2,219_ = 6.26, P = 0.002). NE mink spent significantly more time oriented to ambiguous stimuli than E mink did; for other stimulus types, the pattern was similar but the differences between housing conditions were not statistically significant according to Tukey's HSD tests (see Figure 2). There was also an interaction between stimulus type and stimulus number (i.e. order of presentation; F_2,217_ = 43.1, P<0.0001). However, this resulted from an interaction with housing for stimuli classed as rewarding (F_1,20_ = 8.19, P = 0.010): mink spent less time oriented to the second apparently-rewarding stimulus than the first, and this habituation-like effect was stronger in E mink. Caution is required in interpretation since the second stimulus in this category may not have actually been rewarding, as discussed above. For latency to make contact, housing treatment had a significant main effect only: for all stimuli, NE mink had shorter latencies than E mink did (F_1,28_ = 10.3, P = 0.003; Figure 1). Results for total time spent in contact with stimuli were similar to those for orientation: there was a housing by stimulus type interaction (F_2,189_ = 4.53, P = 0.012), with NE mink spending longer than E mink in contact with all stimuli, but significantly so for ambiguous stimuli only (according to Tukey's HSD tests; see Figure 3). The dependent variables in these tests co-varied significantly. Latency to make contact with the stimulus was inversely correlated with duration of contact, as expected since the two are non-independent (F_1,194_ = 35.9, P<0.0001). It was also inversely correlated with duration of orientation (F_1,218_ = 23.2, P<0.0001).

Results from the second set of analyses, used to confirm that these housing effects held true when the stimuli were not divided into assigned categories, were very similar. Housing treatment had a significant main effect, with apparent interest being higher among NE mink for all three dependent variables: their latencies to make contact were shorter (F_1,193_ = 28.2, P<0.0001), they spent more time oriented to the stimuli (F_1,207_ = 21.9, P<0.0001), and more time in contact with them (F_1,181_ = 36.0, P<0.0001). For time oriented only, this effect differed between specific stimuli (F_9,207_ = 2.40, P = 0.013); however, this appeared to be due to a ceiling effect for the brush only, to which mink in both treatments showed equal, very high orientation.

#### Interest in stimuli: consumption

**Figure 4:**
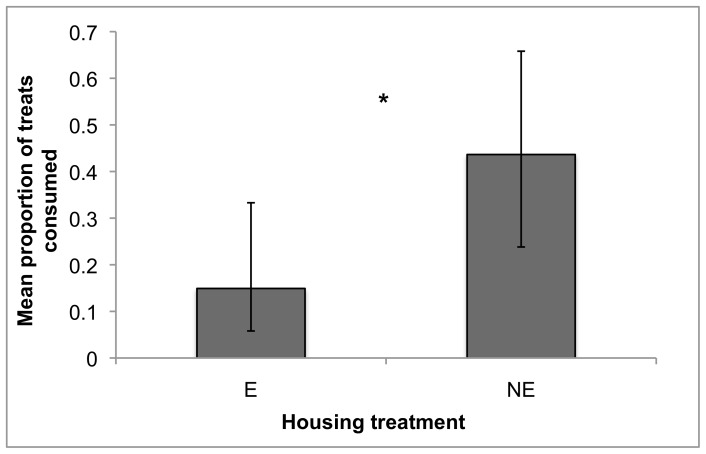
Proportion of treats consumed by housing treatment. Data are back-transformed least squares means across all three treat types, with error bars indicating confidence intervals. * indicates that there is a significant difference between treatments at the α = 0.05 level. A version of this figure based on earlier statistical models was included in Meagher [81].

In the treat consumption tests, NE mink ate a higher proportion of the treats offered than E mink did (F_1,25_ = 4.85, P = 0.037; Figure 4). The proportion of the regular diet that was eaten, in contrast, did not differ between housing treatments, nor was it a significant predictor of treats consumed (both P>0.10).

#### Correction for multiple tests of interest in stimuli

For these tests of effects of housing on apparent interest in stimuli, the appropriate significance level determined for controlling the false discovery rate was P≤0.0043. All of the above results meet this stricter criterion except the housing effect on treat consumption, and the housing by stimulus type effect on duration of contact. However, the main effect of housing on contact was statistically significant even using this criterion (F_1,30_ = 15.0, P = 0.0005), with NE mink spending more time in contact with stimuli overall. Likewise, in the analyses omitting stimulus category, the main effect of housing on interest remained significant after this correction, but the interaction between treatment and stimulus on orientation became non-significant.

### Behavioural correlates of interest indicators

Statistical details of these analyses are presented in Table 4. In brief, lying awake showed positive relationships with exhibited interest in stimuli in several tests, although this was not consistent across stimulus types or across housing treatments. Thus, it had an inverse correlation with latency to make contact for rewarding stimuli (i.e. lying awake frequently was linked to shorter latencies to make contact), but not for ambiguous or aversive stimuli, and a positive correlation with contact duration for aversive stimuli, but not ambiguous or rewarding ones. For duration of orientation, there was an interaction with housing treatment for ambiguous stimuli; however, a split analysis showed no statistically significant relationship in either housing condition, although by inspection, it appeared that mink that spent more time lying awake also spent more time oriented to ambiguous stimuli in E housing. By contrast, there was a trend for more time lying awake to correlate with briefer orientations to rewarding stimuli. In the analyses using overall least square means rather than ones from data split by stimulus category, the results were similar: there were trends for spending more time lying alert to be associated with greater interest in all stimuli. Finally, there was a trend for lying awake to predict more treat consumption in positive trend in E mink only.

**Table 4:** Correlations between interest in stimuli and other behaviour patterns hypothesized to reflect boredom.

Measure	Aversive	Ambiguous	Rewarding	Overall
***Lying awake***				
Latency^1^	n.s.	n.s.	**Negative:** F_1,25_ = 7.21, P = 0.013	***Negative:*** * F_1,26_ = 3.39, P = 0.077*
Duration oriented	n.s.	**E only, ** ***positive:*** * F_1,10_ = 3.35, P = 0.097*; NE: n.s.; Lying*housing: F_1,24_ = 5.00, P = 0.035	n.s.	**E only, ** ***positive:*** * F_1,11_ = 4.35, P = 0.061*; NE: n.s.; Lying*housing: F_1,25_ = 7.68, P = 0.010
Duration in contact^2^	n.s.	n.s.	n.s.	n.s.
Treat consumption^3^	N/A	N/A	**E only, ** ***positive:*** * F_1,7_ = 4.61, P = 0.069*; NE: n.s.; Lying*housing: F_1,19_ = 4.44, P = 0.049	N/A
***Scrabbling***				
Latency	n.s.	n.s.	n.s.	n.s.
Duration oriented	n.s.	n.s.	n.s.	n.s.
Duration in contact	n.s.	n.s.	n.s.	n.s.
Treat consumption^3^	N/A	N/A	n.s.	N/A
***Locomotor stereotypic behaviour (LSB)***				
Latency	n.s.	n.s.	n.s.	n.s.
Duration oriented	**E only, ** ***negative:*** * F_1,11_ = 3.85, P = 0.076*	n.s.	**Negative:** F_1,25_ = 5.49, P = 0.027	n.s. within each treatment
	LSB*housing: F_1,25_ = 6.86, P = 0.015			LSB*housing: F_1,25_ = 4.62, P = 0.041
Duration in contact	**E only, negative:** F_1,11_ = 5.72, P = 0.036	n.s.	**Negative:** F_1,26_ = 4.25, P = 0.049	**E only, ** ***negative:*** * F_1,11_ = 3.90, P = 0.074*
	LSB*housing: F_1,25_ = 8.29, P = 0.008			LSB*housing: F_1,25_ = 4.59, P = 0.042
Treat consumption^3^	N/A	N/A	n.s.	N/A

Durations were least square mean totals for 5-min tests. n.s. = not significant (P>0.05). Italics indicate that a result had 0.05<P<0.10, presented for split analyses only.

1 Log transformation applied;

2 Squared;

3 Logit transformation applied.

Scrabbling showed no significant relationships with interest in stimuli. High levels of locomotor SB, by contrast were associated with low levels of interest in stimuli where there were significant correlations – opposite to the pattern expected if this SB indicated boredom. This was true for aversive and rewarding stimuli only, as indicated by the duration of orientation and the duration of contact; for aversive stimuli, the effect approached statistical significance in E mink only (see Table 4 for details). Again, results were similar in analyses not split by stimulus type: scrabbling bore no relationship to interest in stimuli, while locomotor SB tended to be associated with decreased interest by some measures.

The false discovery rate control procedure indicated that a critical p-value of 0.002 should be used in assessing the relationships between spontaneous behaviour and interest in stimuli in the stimulus tests. None of the above findings met this stricter criterion, and thus replication is required.

## Discussion

The findings were not consistent with apathy or anhedonia: compared to E mink, NE mink were no less responsive to stimuli in general or to rewards in particular. In contrast, the data were broadly consistent with boredom, with NE mink exhibiting more interest in all stimuli presented to them than E mink did. NE mink had significantly shorter latencies to approach all stimuli, regardless of stimulus type. They spent more time oriented to and more time in contact with all the stimuli, an effect that reached significance for ambiguous stimuli. They ate significantly more treats in the 15-minute test period. All of these effects proved robust, holding true even using strict controls for multiple statistical testing, with the exception of the difference in treat consumption. They also held true when stimuli were treated as an array rather than divided into pre-set categories, further suggesting that this housing effect is a general one, holding true across diverse stimuli. Spending time lying awake in the home cage, inert but with the eyes open, also co-varied positively with enhanced interest in the test stimuli, as predicted if this behaviour pattern reflected boredom. Locomotor SB, in contrast, showed inverse correlations with interest in the stimuli, contrary to this prediction and suggesting that this activity may even have a negative relationship with boredom-like states. Stereotypic scrabbling showed no relationship with these indicators of boredom. These last effects all need replicating, however, since there is some risk that they were Type I errors.

The insights yielded by these data were critically dependent on using a diverse array of stimuli. Indeed, this is the first experiment of its kind to systematically investigate how enrichment affects responses across stimulus types. (Previous studies examined responses to only one or a few stimuli, e.g. [60], which were typically expected to be neutral). Our *a priori* categorization of the stimuli was validated by the increased incidence of fear-related behaviour when presented with stimuli classed as aversive. The distinction between rewarding and aversive stimuli was less clear. The possible misclassification of one or two specific stimuli (female faeces and bobcat urine) had little effect on the results or their interpretation, however, because the mink appeared to demonstrate a boredom-like state rather than apathy or depression. Thus, whether or not the stimuli were divided into categories, NE mink showed increased interest across the whole diverse range: the central prediction for this psychological state. Furthermore, their increased consumption of food treats shows that this extended to unambiguously rewarding stimuli as well as aversive and ambiguous ones, so allowing us to reject the hypothesis that they were suffering from anhedonia. Future research, however, should include *a priori* better-validated classifications of rewarding/aversive/neutral stimuli for a given species, to help in selecting a manageable number of stimuli while still ensuring the diversity required for this type of experiment. As we have shown, exploratory responses may not help here. However, rewarding, neutral and aversive stimuli could be differentiated based on modulation of the startle response, which is attenuated by presenting a rewarding stimulus but exacerbated by aversive ones [61].

Using a diverse array of stimuli in our comparison of E and NE mink also allowed us to consider and eliminate several possible alternative explanations for the observed housing effects. In previous research examining how housing influences responses to stimuli, increased fear in impoverished housing, which would increase avoidance of novel stimuli, was typically a potential confound. The current results cannot be explained by increased fearfulness in NE mink, since they showed no more overt signs of fear than E mink even when faced with fear-provoking stimuli; indeed, they exhibited shorter rather than longer latencies to make contact with all stimuli, including these aversive ones. We could also demonstrate that hunger was not responsible for the increased treat consumption of NE mink (since amount of the regular diet eaten was unaffected and did not predict treat consumption), so validating the use of these tests as a measure of specific response to reward. Finally, previous work has indicated that non-enriched housing can increase motivations for rewarding stimuli specifically (e.g. [62]), an effect reflecting stress-induced sensitization of a reward pathway in the brain, the mesoaccumbens dopamine system (reviewed by [63], [64]). The same process could perhaps have been invoked to explain the increased consumption of food treats by NE mink, but it is incompatible with our demonstration that this is part of a broader pattern of increased interest in all stimuli: ambiguous and aversive ones too. Thus, boredom in NE animals is the most parsimonious explanation for the pattern of results. This methodological approach would thus seem a very useful one, which now could be applied to other species and housing conditions.

To date, studies of other species have found conflicting results regarding whether the lack of enrichment increases exploratory behaviour [5], [60], [65], as in our mink, or instead decreases it ([66], cited in [67]; [68], [69]). These differences are hard to interpret because the studies used a narrower range of stimuli than were tested here. As a result, they could reflect the stimulus-specific housing effects on fear or the stress-sensitization of reward systems outlined above. However, it is also possible that they reflect differences between species, life stages and/or housing systems in the non-stimulus-specific effects that are our focus: thus in whether non-enriched subjects develop boredom, or instead depression or apathy. We therefore suggest that responses to a diverse array of stimuli, as assessed here, should now be assayed in a range of species and housing systems to screen for such effects more widely. Such data could also be used to test specific hypotheses, both fundamental and applied. In mink, for example, they could investigate whether boredom in non-enriched animals is reversed rapidly by the provision of enrichments (unlike apathy or depression, as we discuss below); whether strengths of motivations to interact with environmental enrichments (e.g. assessed using weighted doors that allow mink to access enrichments by paying a price, cf. [70]) are predicted by prior levels of boredom; and whether simpler enrichments, feasible for use on commercial mink farms, successfully reduce boredom. In other animals, our technique could be used to test long-standing hypotheses that generalist, opportunistic species (e.g. raccoons or wolves) are prone to boredom in captivity [71], [72], and whether intelligent animals (e.g. apes or dolphins) are particularly at risk [73]. More fundamentally, they could also reveal whether animal boredom shares neurophysiological correlates with those newly discovered for ‘sensation-seeking’, a human trait characterized by strong motivations to seek pleasure and danger [74], [75]. Where such data instead reveal depression or apathy, they could help investigate whether these more severe responses to impoverished environments reflect differences between species or life stages tested, as discussed above; differences in whether the enrichment used reduces fear; or instead differences in the duration of the impoverished housing, with chronic boredom possibly developing into apathy and/or depression over time ([18]; humans: [21]). Finally, our technique could also be used to test whether enrichment is more successful at improving welfare if introduced when the animals are bored rather than when they have become apathetic or depressed [18].

Our other main finding was that one spontaneous home-cage behaviour, lying awake and inactive, was a potential marker of boredom in mink, since it correlated with the elevated interest in stimuli induced by impoverished housing. This result potentially explains why lying awake is elevated in non-enriched housing but not associated with increased fearfulness or cortisol levels [44], since boredom is a negative affective state but not necessarily associated with increased arousal [28], [30]. The relationships between lying awake and heightened interest in stimuli were detected only in the enriched group in the current experiment on some measures, which was unexpected given that these mink seemed to experience lower levels of boredom. However, this is likely due to the very limited variance in responses in the non-enriched group: NE mink all seemed uniformly very interested in stimuli, creating a ceiling effect that made it difficult to detect a correlation. Such effects clearly now need further investigation, not least as our controls for multiple testing highlighted them as potential Type I errors. However, this finding is intriguing given that forms of alert inactivity have likewise been hypothesized to indicate boredom in other species (e.g. [1], [32] on pigs and rabbits). It is also worth pursuing with future research since lying awake can be assessed quite easily, without a prolonged testing regime.

The potential relationship found between boredom and SB likewise needs treating with caution, although again it deserves future investigation. While scrabbling did not co-vary with responses to stimuli in any tests, locomotor SB showed significant correlations with exhibited interest in the opposite direction to that predicted: the most exploratory, hence arguably bored, mink exhibited the *least* locomotor SB. Locomotor SB in mink includes pacing and other SBs typical of caged Carnivora. Does this support the hypothesis advanced by Kiley-Worthington [76], that SB can be a method of coping with boredom induced by under-stimulating environments, perhaps by providing self-stimulation? Is locomotor SB thus the type of SB that Mason and Latham [77] suggested acts as a ‘do-it-yourself’ enrichment? Such hypotheses do have some support from humans with autism, for whom the opportunity to perform stereotypic behaviour can serve as a positive reinforcer [78], and so these findings are intriguing. Nonetheless, the evidence from the current experiment is only correlational, and so cannot provide causal evidence that SB is helping these individuals to cope; it may instead be linked to a decreased ability to respond appropriately to novel stimuli (see [31], [79]) for reasons unrelated to boredom. These effects were also rather weak statistically: they now require confirmation in replicate studies.

Overall, this study provides a first step towards operationalizing boredom in non-human animals. Although we cannot yet determine with certainty whether the subjective experience of the animals is similar to that of humans who self-report feeling bored, their behaviour was consistent with that state. Such means of operationally defining boredom for non-humans so that it can be quantified are very much needed, since reducing boredom is often a stated aim of enrichment (e.g. [80]), and yet to date we have had no means of judging its success at achieving that aim. As well as being aversive, boredom has a variety of negative consequences (e.g. health problems) in humans [20], and thus truly deserves further study in other animals. The results of this study also cautiously provide evidence contrary to the hypothesis that locomotor SB reflects boredom on an individual level; in mink and perhaps other captive carnivores, it may be induced by boring environments, but it may actually help to alleviate that boredom. Lying awake for a large portion of the day, in contrast, merits further study as a possible indicator of boredom in both this, and other, species.
